# Supplementary material for: Resolving the full spectrum of human genome variation using Linked-Reads
Source: Genome Res. 2019 Apr;29(4):635–45. doi: 10.1101/gr.234443.118 (PMC6442396; doi:10.1101/gr.234443.118)
Supplement: Supplemental Material [file supp_gr.234443.118_Supplemental_longranger-2.2.zip › longranger-2.2.tar.bz2!/lr2.2-public/lib/stan/cmdstan/stan/doc/api/html/bare__type__grammar__def_8hpp.html]

Stan: src/stan/gm/grammars/bare\_type\_grammar\_def.hpp File Reference


|  |  |
| --- | --- |
|  | Stan  2.5.0  probability, sampling & optimization |


- Main Page
- Related Pages
- Namespaces
- Classes
- Files

- File List
- File Members

All Classes Namespaces Files Functions Variables Typedefs Enumerations Enumerator Friends Macros Pages

- src
- stan
- gm
- grammars

Namespaces |
Functions

bare\_type\_grammar\_def.hpp File Reference

`#include <boost/spirit/include/qi.hpp>`  
`#include <boost/lexical_cast.hpp>`  
`#include <boost/fusion/include/adapt_struct.hpp>`  
`#include <boost/fusion/include/std_pair.hpp>`  
`#include <boost/config/warning_disable.hpp>`  
`#include <boost/spirit/include/qi_numeric.hpp>`  
`#include <boost/spirit/include/phoenix_core.hpp>`  
`#include <boost/spirit/include/phoenix_function.hpp>`  
`#include <boost/spirit/include/phoenix_fusion.hpp>`  
`#include <boost/spirit/include/phoenix_object.hpp>`  
`#include <boost/spirit/include/phoenix_operator.hpp>`  
`#include <boost/spirit/include/phoenix_stl.hpp>`  
`#include <boost/spirit/include/support_multi_pass.hpp>`  
`#include <boost/tuple/tuple.hpp>`  
`#include <boost/variant/apply_visitor.hpp>`  
`#include <boost/variant/recursive_variant.hpp>`  
`#include <boost/spirit/include/version.hpp>`  
`#include <boost/spirit/include/support_line_pos_iterator.hpp>`  
`#include <stan/gm/ast.hpp>`  
`#include <stan/gm/grammars/bare_type_grammar.hpp>`  
`#include <stan/gm/grammars/whitespace_grammar.hpp>`

Go to the source code of this file.

|  |  |
| --- | --- |
| Namespaces | |
|  | stan |
|  | Probability, optimization and sampling library. |
|  | |
|  | stan::gm |
|  | Directed graphical model compiler. |
|  | |

|  |  |
| --- | --- |
| Functions | |
|  | BOOST\_FUSION\_ADAPT\_STRUCT (stan::gm::expr\_type,(stan::gm::base\_expr\_type, base\_type\_)(size\_t, num\_dims\_)) |
|  | |

## Function Documentation

|  |  |  |  |
| --- | --- | --- | --- |
| BOOST\_FUSION\_ADAPT\_STRUCT | ( | stan::gm::expr\_type | , |
|  |  | (stan::gm::base\_expr\_type, base\_type\_)(size\_t, num\_dims\_) |  |
|  | ) |  |  |


---

[ Stan Home Page ]

*© 2011–2014,
Stan Development Team.*
